# Perspective: a stirring role for metabolism in cells

**DOI:** 10.15252/msb.202110822

**Published:** 2022-04-01

**Authors:** José Losa, Simeon Leupold, Diego Alonso‐Martinez, Petteri Vainikka, Sebastian Thallmair, Katarzyna M Tych, Siewert J Marrink, Matthias Heinemann

**Affiliations:** ^1^ Molecular Systems Biology Groningen Biomolecular Sciences and Biotechnology Institute University of Groningen Groningen The Netherlands; ^2^ Molecular Dynamics Groningen Biomolecular Sciences and Biotechnology Institute University of Groningen Groningen The Netherlands; ^3^ Chemical Biology Groningen Biomolecular Sciences and Biotechnology Institute University of Groningen Groningen The Netherlands; ^4^ Present address: Frankfurt Institute for Advanced Studies Frankfurt am Main Germany

**Keywords:** active matter, enhanced diffusion, Gibbs energy, metabolism, regulation, Metabolism

## Abstract

Based on recent findings indicating that metabolism might be governed by a limit on the rate at which cells can dissipate Gibbs energy, in this Perspective, we propose a new mechanism of how metabolic activity could globally regulate biomolecular processes in a cell. Specifically, we postulate that Gibbs energy released in metabolic reactions is used to perform work, allowing enzymes to self‐propel or to break free from supramolecular structures. This catalysis‐induced enzyme movement will result in increased intracellular motion, which in turn can compromise biomolecular functions. Once the increased intracellular motion has a detrimental effect on regulatory mechanisms, this will establish a feedback mechanism on metabolic activity, and result in the observed thermodynamic limit. While this proposed explanation for the identified upper rate limit on cellular Gibbs energy dissipation rate awaits experimental validation, it offers an intriguing perspective of how metabolic activity can globally affect biomolecular functions and will hopefully spark new research.

## Graphical abstract

Recent findings suggested that metabolism is governed by a limit on the rate at which cells can dissipate Gibbs energy. This Perspective proposes a new mechanism of how metabolic activity and the Gibbs energy released in metabolic reactions could globally regulate biomolecular processes.

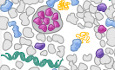

## A new hypothesis explaining how metabolic activity affects biomolecular functions

Metabolism and other cellular functions are controlled by a plethora of regulatory mechanisms. However, in recent years, it has become clear that metabolism is not only subject to regulation, but metabolic cues themselves can also regulate other cellular functions (Haas *et al*, 2016; Ryan *et al*, 2018; Zhu & Thompson, 2019; Orozco *et al*, 2020). In most of these cases, altered levels of metabolites trigger regulatory action, for instance, by binding to transcription factors (Kochanowski *et al*, 2017; Lempp *et al*, 2019), or by allosteric interactions with enzymes or other biomolecules (Hackett *et al*, 2016; Sander *et al*, 2019). In this Perspective, we propose that besides these very specific and well‐studied metabolite‐dependent regulation mechanisms, there might be an additional global mechanism of how an active metabolism could affect essentially all biomolecular functions in a cell.

**Figure 1:**
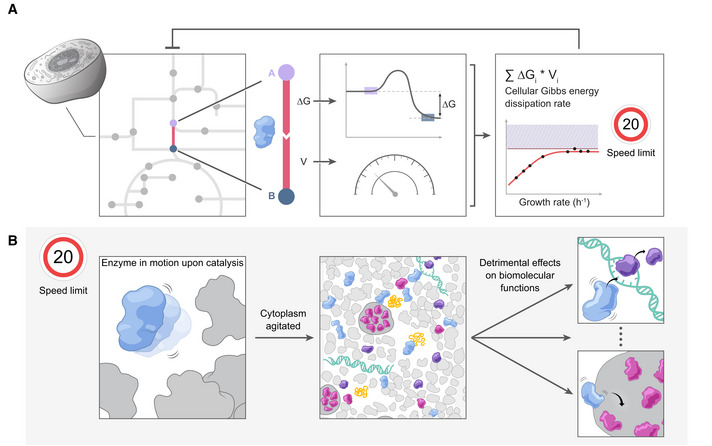
Cellular Gibbs energy dissipation rate has an upper limit and may be explained by metabolism‐induced molecular motion (A) A cell encompasses a large number of interconnected chemical reactions, forming a metabolic network (left panel); each reaction is catalyzed by an enzyme and is characterized by two parameters: its Gibbs energy of reaction, Δ*G*, and its flux, *v* (middle panel). The product of the Gibbs energy of reaction and the flux is the Gibbs energy dissipation rate. When this parameter is summed across all the reactions in the metabolic network, the cellular Gibbs energy dissipation rate is obtained. The cellular Gibbs energy dissipation rate has units of J/h, or J/gDW/h if normalized to the cell dry biomass. We previously found that this parameter reaches an upper limit, at moderately high growth rates/substrate uptake rates (Niebel *et al*, 2019) (right panel). Constrained by this upper limit, cells that have reached the “plateau” may still achieve increased growth rates provided that metabolic fluxes are redirected towards reactions that dissipate less Gibbs energy. The interplay between the upper limit and metabolism is represented by the negative feedback arrow. (B) To explain the existence of this limit, we propose that catalysis leads to enhanced enzyme motion (left panel). In the crowded intracellular environment (middle panel), excessive motion will ultimately cause detrimental effects on biomolecular functions (right panel).

This proposed mechanism stems from our previous work (Niebel *et al*, 2019), in which we proposed that a thermodynamic limit could govern cellular metabolism, *i.e*., that a thermodynamic limit could determine the intracellular metabolic flux distribution. In brief, cellular metabolism consists of many different chemical reactions (Fig 1A, left part). Each of these reactions carries a certain flux (*v*) and exhibits a particular Gibbs energy of reaction (∆*_r_G*) (Fig 1A, middle part, for further explanation of terms cf. Table 1). The product of a reaction’s flux and Gibbs energy defines the rate at which Gibbs energy is dissipated in this reaction. If the Gibbs energy dissipation rates of all the metabolic reactions occurring in a cell are summed up, we obtain the cellular Gibbs energy dissipation rate. In our study (Niebel *et al*, 2019), we uncovered that a limit might exist on this rate (Fig 1A, right part). Once the glucose uptake rate is high and the limiting rate of Gibbs energy dissipation is reached, *Saccharomyces cerevisiae* and *Escherichia coli* start to excrete ethanol and acetate, respectively, in a behavior which is known as aerobic fermentation or overflow metabolism. Remarkably, despite the fact that both organisms have largely different cell volumes, the value of this limit was found to be in the same order of magnitude (when normalized by the cellular dry weight), suggesting that it does not simply scale with cell morphology, but is rather an intrinsic property of the organisms. When we used the identified limit of the Gibbs energy dissipation rate as a constraint in flux balance analysis simulations (Orth *et al*, 2010) while maximizing biomass production (*i.e*., growth rate), we obtained excellent predictions of metabolic phenotypes. These predictions included the shift from a respiratory towards a fermentative metabolism at increased glucose uptake rates (as the latter provides a less dissipative way for metabolizing carbon), maximal growth rates on various nutrients (including in complex media), intracellular flux distributions and even predictions of changes in metabolite concentrations (Niebel *et al*, 2019). While using an additional constraint in flux balance analysis simulations is not a new approach (see Box 1), the excellent agreement of these predictions with experimental data, and their broad scope, suggested that an upper limit on the cellular Gibbs energy dissipation rate could indeed exist and that this limit may govern cellular metabolism and growth.

**Table 1:** Key terminology.

Steady‐state	State in which the parameters of the system (metabolite concentration, reaction fluxes, Gibbs energy dissipation rate) remain constant over time. May be achieved even under *non*‐equilibrium conditions
Equilibrium	State in which the thermodynamic driving force (e.g., ∆*_r_G*) is null, hence the net fluxes (e.g., metabolic rate, Gibbs energy dissipation rate) are likewise null. In contrast, *non‐*equilibrium is characterized by a non‐zero driving force, and may or may not be at steady state
Gibbs energy of reaction (∆*_r_G*)	Thermodynamic driving force associated with a chemical reaction. Negative values of this parameter (∆*_r_G* < 0) are associated with thermodynamic feasibility of the reaction to proceed in the forward direction. It is a function of the momentary concentration of substrates and products (*c_i_*), affected by their stoichiometric coefficients (*S_i_*), in the reaction: $\Delta_{r}G=\Delta_{r}G^{0}+RT\cdot \sum_{i}S_{i}\ln\left(c_{i}\right)$, with $\Delta_{r}G^{0}$ being the Gibbs energy of reaction under standard conditions (a tabulated value)
Metabolic flux (*v*)	Rate at which a metabolic reaction occurs, measuring the amount of substrate(s) consumed or product(s) formed per unit of time
Gibbs energy dissipation rate (*g*)	Defined for a *single reaction* as the product of its flux, *v*, and the associated Gibbs energy of reaction, ∆*_r_G*, thus: $g=v*\Delta_{r}G$. It is non‐zero for any reaction that is out of equilibrium
*Cellular* Gibbs energy dissipation rate	Same as above, applied to *a whole cell*, as represented by a macrochemical equation (“*substrates→ biomass + byproducts*”). It can be obtained by summing the Gibbs energy dissipation rates of all reactions taking place in the cell
Thermal Brownian motion	Random, diffusive motion that a small particle undergoes in a medium due to temperature. The higher the temperature, the faster the particle moves, so that its diffusion coefficient, *D*, is higher. At thermal equilibrium, the value of the diffusion coefficient can be estimated from the Stokes‐Einstein equation, D=c*T/η*R, where *c* is a constant, *T* is the temperature, *R* is the particle radius and *η* is the fluid viscosity
Newtonian fluid	A fluid with viscosity which is independent of the shear rate (*i.e*., rate at which the fluid is deformed)
Non‐reciprocal conformational changes	Conformational changes (of proteins, for example) which are asymmetric with respect to time. The initial conformation can be reached by a “path” which does *not* imply the mere reversal of a conformational change
Multivalent interactions	Interactions established between two molecules, spread out over multiple regions of each of these molecules
Supramolecular structure	A loose aggregate of proteins and other macromolecules, held together by multivalent interactions
Phase separation	Phenomenon by which homogeneously mixed molecules separate into distinct, coexisting, liquid phases. While one of the phases is depleted of some of those components, the other is enriched

Brief explanation of some of the most relevant terms used in the text.

**Box 1. Comparison of the upper limit on Gibbs energy dissipation rate with alternative constraints on metabolism**

The cellular Gibbs energy dissipation rate is the sum over all fluxes (*v*) in a metabolic network, where each flux is weighed by the corresponding Gibbs energy of reaction (Δ*_r_G*) (Equation 1). Imposing a limit on the cellular Gibbs energy dissipation rate in flux balance analysis models means that an upper constraint, $g_{diss^{\lim}}$, is imposed on this variable:

(1) $$\sum_{i}v_{i}*\Delta_{r}G_{i}\leq g_{diss^{\text{lim}}}.$$

From a mathematical point of view, the upper limit on the cellular Gibbs energy dissipation rate is analogous to other constraints that have been used in flux balance analysis to account for “resource allocation”, e.g., in terms of total amount of proteins in the cell, macromolecular crowding, or membrane occupancy (Basan *et al*, 2015; Vazquez & Oltvai, 2016; Szenk *et al*, 2017; Elsemman *et al*, 2022). All such constraints resemble a weighted sum of fluxes. The rationale here is that each reaction comes with a certain “cost” in terms of the given resource (e.g., fraction of the cell proteome, volume, or surface area occupied by the enzymes; represented by *w*), where the sum of the individual costs must not surpass the limited capacity of the cell, *C*:

(2) $$\sum_{i}v_{i}*w_{i}\leq C.$$

The structural similarity of these constraints (thermodynamic‐ or resource allocation‐based) leads to a similar outcome in the predictions from the models: in either case, overflow metabolism (*i.e*., simultaneous use of respiratory and fermentative pathways at high substrate uptake rates) is predicted (de Groot *et al*, 2020). Remarkably, the similarity between the constraints may extend beyond their analogous mathematical formulations. For example, it is possible that the thermodynamic constraint is intrinsically related with the proteome allocation constraint. On the one hand, the biological strategy evolved to cope with reactions that involve a large Δ*_r_G* (e.g., in respiration, where the substrates are fully oxidized) is characterized by a splitting of the overall reaction into multiple steps, each associated with an enzyme. As a result, highly dissipating pathways are also pathways that have a larger cost in terms of resources (namely proteins). On the other hand, the causality may also go in the reverse direction: if proteome constraints limit the number of proteins available in a certain pathway, then metabolic flux is decreased, and the overall Gibbs energy dissipation rate in that pathway is similarly reduced (because the dissipation rate is the product of flux and Gibbs energy of reaction).

Future work is needed to further investigate the molecular basis of these constraints and their putative connection. While the “resource allocation”‐based constraints are intuitively apprehended, the constraint on the cellular Gibbs energy dissipation rate is both more challenging to explain mechanistically and more difficult to test experimentally. It is a goal of this perspective to put forward mechanistic ideas and intuition about the thermodynamic constraint, thereby potentially opening a new view on how metabolic activity could globally affect biomolecular functions and ultimately “constrain” metabolism.

While the observation that cellular metabolism might be governed by a thermodynamic limit is intriguing, it also triggers a new question: what could be the molecular mechanism that underlies this upper limit on the cellular Gibbs energy dissipation rate? Or, in other words, why would cells—which, according to Erwin Schrödinger, need to *“free [themselves] from all the entropy [they] cannot help producing while alive”* (Schrödinger, 1944)—be limited by the rate at which they can do so? Here, dwelling on this question, and pulling together fragmented pieces of evidence from diverse research fields, we developed a bold hypothesis to explain this limit. This hypothesis might explain the physiological behavior of cells from the molecular level of enzymes. Specifically, we hypothesize that during their catalytic action, metabolic enzymes use part of the released Gibbs energy to increase their motion, *i.e*., to perform work (Fig 1B, left part). This then leads to increased movement of biomolecules in cells (Fig 1B, middle part), which can, in turn, affect regulatory and biomolecular processes, with some being favored and others disfavored, by the increasing rates of collision (Fig 1B, right part). If regulatory processes such as transcription or translation are dependent on molecular motion, a feedback loop can be established, where metabolic activity (and thus Gibbs energy dissipation) is kept under control, resulting in a maximal limit on the cellular Gibbs energy dissipation rate.

In this perspective article, we start by asking what the global effects of the cellular Gibbs energy dissipation could be. Second, we explore whether enzymes can perform work during catalysis. Third, we ask what the consequences of increased molecular motion could be for a cell. Overall, this article offers an intriguing perspective on how metabolic activity could globally affect biomolecular functions.

## What could be the global effects of cellular Gibbs energy dissipation?

In a first instance, one could interpret the upper limit on the cellular Gibbs energy dissipation rate as a limit on the heat transfer rate from the cell to its surroundings. Under this assumption, increased metabolic activity, and thus heat production, could lead to increased temperature in the cell or its compartments (e.g., mitochondria), which could potentially damage proteins or other macromolecules, or have other detrimental effects on cellular processes. Supporting this idea, intracellular temperature measurements using a variety of thermosensors (Zohar *et al*, 1998; Yang *et al*, 2011; Okabe *et al*, 2012; Kiyonaka *et al*, 2013; Takei *et al*, 2014; Arai *et al*, 2015; Chrétien *et al*, 2018; Savchuk *et al*, 2019) suggested that there are temperature differences between cellular compartments. The mitochondrion, in particular, was suggested to have a temperature higher than that of the cytoplasm (Okabe *et al*, 2012; Chrétien *et al*, 2018). Notably, the mitochondrion is a compartment where a high Gibbs energy dissipation rate is expected, with the respiratory chain contributing 50% to the cellular Gibbs energy dissipation rate under certain conditions (Niebel *et al*, 2019).

However, the conclusions from experiments with thermosensors have been questioned (Baffou *et al*, 2014). Assuming steady‐state conditions and neglecting entropic effects (so that the change of Gibbs energy equals an enthalpy change, *i.e*., *all* of the Gibbs energy dissipated is converted into heat), the temperature in the center of a yeast cell, for example, would only increase by 10^−5^ K compared to the temperature in the environment. The reported temperature measurements cannot be explained even when accounting for (i) spatially confined heat release (e.g., all heat is released in the mitochondria), (ii) a temporal variation of the release (all heat is released in a temporal burst), or (iii) a finite thermal conductivity of membranes (considering an insulator effect of the cell membrane) (Baffou *et al*, 2014). This discrepancy has become known as the “10^5^ gap” (Suzuki *et al*, 2015). While the gap may in part be due to the sensitivity of the thermosensors to molecular events in their surroundings (Lane, 2018), and as recently shown, introducing more accurate estimates of the thermal conductivity in a cell may help reduce this gap (Sotoma *et al*, 2021)), the difference between predicted and measured temperature values is still far from being negligible (Suzuki & Plakhotnik, 2020). Overall, limited heat transfer, resulting in increased and detrimental intracellular temperature, cannot explain the observed limit on the Gibbs energy dissipation rate.

Alternatively, Gibbs energy released during metabolic activity could be used to perform mechanical work, and cells could be limited by the amount of “work” they can “withstand”. Along these lines, one could hypothesize that the Gibbs energy released during enzyme catalysis is harnessed to increase the motion of the catalyzing enzymes. Such increased motion may potentially be a result of enzymes undergoing self‐propulsion during their catalytic action, by the enzymes breaking free from a supramolecular structure, or a combination of both. If indeed Gibbs energy is harnessed as work, one would expect higher diffusion coefficients in metabolically active cells. It is conceivable that too much intracellular molecular motion is not compatible with proper cellular functioning, thus establishing a limit on the cellular Gibbs energy dissipation rate. This is the idea we want to put forward here.

**Figure 2:**
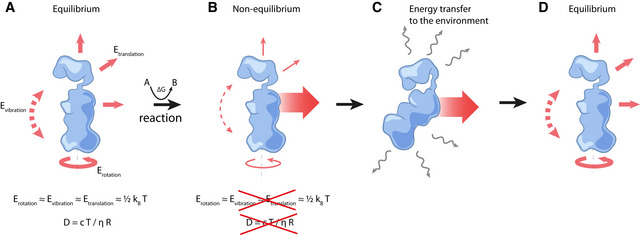
Increased molecular motion can occur at hardly increased temperature (A) According to the equipartition theorem, the average energy of all degrees of freedom (rotation, vibration, and translation) is the same, and equal to $\frac{1}{2}k_{B}T$. Under these conditions, the diffusion coefficient can be estimated from the Stokes–Einstein equation, and the enzyme is said to undergo thermal Brownian motion (see definition in Table 1). (B) For an enzyme driven out of equilibrium by the Gibbs energy released during catalysis, the equal distribution of energy among the various degrees of freedom no longer applies, and the diffusion coefficient may not abide by the Stokes–Einstein equation, potentially being larger than the value predicted by this equation. (C) As it moves, the enzyme also dissipates energy to its surroundings as heat, which is swiftly transferred to the environment. (D) The loss of energy brings the enzyme back to its initial equilibrium state with the local surroundings.

However, critical readers may wonder how no increase in intracellular temperature and increased molecular movement can be compatible, given that temperature is typically defined as the average kinetic energy of the molecules in a system. As we explain in Box 2 (Fig 2A), these two arguments are not physically inconsistent in the non‐equilibrium situation of a living cell. Thus, increased molecular motion, induced by enzyme catalysis, could indeed occur without significantly increasing intracellular temperature.

**Box 2. In a non‐equilibrium system, increased molecular motion can occur at hardly increased temperature**

In statistical physics, the temperature of a system is defined, at equilibrium, as the average kinetic energy of the ensemble of all molecules it contains. Equivalently, and following the equipartition theorem, the energy of each degree of freedom of the system can also be expressed as a function of temperature, *T*, according to the expression $\frac{1}{2}k_{B}T$, where *k_B_* is the Boltzmann constant. In other words, there is as much kinetic energy associated with the translation of a molecule along any of the three spatial directions as in the rotation or vibration of this molecule (Fig 2A).

Out of equilibrium, which is the condition in which cells operate, however, the equipartition theorem ceases to apply: it is then possible that any one of the degrees of freedom (*i.e*., translation, rotation, vibration) is associated with more energy than the remaining ones (Casas‐Vázquez & Jou, 2003), and the average energy of each degree of freedom (taken across all molecules of the system) is no longer necessarily given by $\frac{1}{2}k_{B}T$ (Fig 2A and B).

As such, when a biochemical reaction, by releasing Gibbs energy, drives the system out of equilibrium, there can be an enhancement in the mobility of enzymes (see the main text for a discussion on the possible mechanisms), above the levels that would be expected based on thermal Brownian motion. That is, the diffusion coefficient increases above the value that is normally obtained for that enzyme under equilibrium conditions. Yet, in this out of equilibrium state, collisions with surrounding molecules inevitably lead to the dissipation of energy in the form of heat (Fig 2B–D). Nevertheless, the efficient heat transfer across the cell (Baffou *et al*, 2014), ensures that this is rapidly removed from the system.

There are indications that Gibbs energy is indeed released during enzyme catalysis and leads to increased intracellular molecular motion. Investigations of the *in vivo* jiggle of chromosomal loci in yeast and bacteria have indicated a correlation between metabolic activity and the diffusion of the tracked loci (Weber *et al*, 2012): when cells were treated with sodium azide and 2‐deoxyglucose, inhibiting the synthesis of ATP, the apparent diffusion coefficient of the observed chromosomal locus decreased by half compared to untreated cells. Cells only treated with sodium azide, allowing for the synthesis of some ATP through glycolysis, exhibited an intermediate phenotype. If the movement of the chromosomal loci were only due to thermal Brownian motion, a linear relationship between the observed diffusion coefficient and temperature would be expected, according to the Stokes‐Einstein relation. However, the observed diffusion coefficient instead showed an exponential relationship with temperature, in agreement with the Arrhenius equation, which describes the influence of temperature on the rate of chemical reactions. These observations suggest that enzyme catalysis, and thus metabolism, may indeed lead to enhanced translational motion of molecules in the cell.

The observation that metabolic activity fluidizes the cytoplasm, which is otherwise in a glass‐like state, provides further evidence that metabolism induces molecular motion inside the cell (Parry *et al*, 2014; Nishizawa *et al*, 2017; Åberg & Poolman, 2021). It has been argued that the fluidity of the cytoplasm is dependent on metabolism‐induced physicochemical changes, namely in cytoplasmic pH (Munder *et al*, 2016), cellular crowding (Joyner *et al*, 2016; Delarue *et al*, 2018), or ATP concentration (Patel *et al*, 2017; Persson *et al*, 2020). Nevertheless, it can also be envisioned that the catalysis‐induced molecular motion, *i.e*., the agitation of the cytoplasm by active enzymes, is capable of fluidizing it (Parry *et al*, 2014), playing a role analogous to that of molecular motors in eukaryotic cells (Lau *et al*, 2003; Guo *et al*, 2014). In fact, molecular dynamics analyses where proteins were subjected to changes in volume have shown that even small changes in the diffusion coefficient of proteins can lead to significant changes in the fluidization state of the cytoplasm (Oyama *et al*, 2019). Further evidence for increased molecular motion in cells comes from statistical thermodynamic analyses of a simplified model of translation initiation in bacteria. Under the assumptions that mRNA‐ribosome complexes are at equilibrium and that the translation rate is proportional to the number of such complexes, relating the abundance of a fluorescent reporter to the calculated Gibbs energy of the ribosome‐mRNA binding step led to the suggestion that mRNA binding to the ribosome requires a system temperature of more than 1,000 K (Salis, 2011), which is clearly outside the range of life‐permitting values. Yet, translation initiation does occur, and therefore, this discrepancy may potentially be explained by non‐equilibrium effects, such as increased molecular motion. One final example of enhanced motion coupled to enzymatic activity is the observation that membranes become softer, showing undulations akin to those arising from an elevated temperature (up to three‐fold enhancement in effective temperature in case of Ca^2+^‐ATPase), when the membrane‐embedded protein channels are active (Prost & Bruinsma, 1996; Girard *et al*, 2005).

Taken together, there is evidence that in metabolically active cells, molecules move more than would be expected from thermal Brownian motion alone. This is the case even in bacteria, which lack gliding of macromolecules along the cytoskeletal structures. Changes in physicochemical parameters may also contribute to changes in intracellular diffusion rates and gradients of chemical compounds have been implicated in driving convective flows (Ortiz‐Rivera *et al*, 2016; Testa *et al*, 2021). Nevertheless, in the following sections, we argue that the connection between the observed increase in intracellular motion and metabolic activity could be established through enzymes performing work by using the Gibbs energy released during catalysis.

## How could enzymes perform work during catalysis?

If, as indicated by several observations, metabolism plays a role in “stirring up” the cytoplasm, then Gibbs energy released in enzymatic reactions must somehow be transduced into mechanical work. Some archetypal examples of mechanical transduction at the molecular scale include motor proteins, which use Gibbs energy to induce movement along cytoskeletal filaments (e.g., kinesin and myosin) and nucleic acids (e.g., polymerases, topoisomerases and gyrases), or to rotate bacterial flagella and F_0_F_1_‐ATP synthase (Phillips *et al*, 2012; Kolomeisky, 2013; Guo *et al*, 2014). Also in chemistry, it is known that catalytically active asymmetric micro‐/nanoscale objects can self‐propel during catalysis (Ismagilov *et al*, 2002; Qin *et al*, 2017; Zhao *et al*, 2018a; Arqué *et al*, 2019; Sun *et al*, 2019; Luo *et al*, 2020), and that both reactant and solvent molecules can experience increased mobility upon catalysis, even when gas formation and convection are ruled out (Wang *et al*, 2020).

Analogously, there is evidence that metabolic enzymes show motor‐like behavior. For instance, multiple fluorescence correlation spectroscopy (FCS) experiments, in which the diffusion coefficients of fluorescently labeled enzymes are inferred from fluorescence fluctuations, showed enhanced diffusion of free‐swimming or membrane‐bound enzymes when mixed with their substrates. This was shown for enzymes such as urease (Muddana *et al*, 2010; Riedel *et al*, 2015; Jee *et al*, 2018a, 2018b; Ghosh *et al*, 2019), catalase (Sengupta *et al*, 2013; Riedel *et al*, 2015), alkaline phosphatase (Riedel *et al*, 2015; Ghosh *et al*, 2019), fructose bisphosphate aldolase (Illien *et al*, 2017), acetylcholinesterase (Jee *et al*, 2018b), ATPase (Ghosh *et al*, 2019), and hexokinase (Zhao *et al*, 2018b). Likewise, motility increase in membrane‐bound enzymes was observed with single‐particle tracking techniques (Ghosh *et al*, 2019; Song *et al*, 2021). In some cases, the change in diffusion was explained by conformational changes upon substrate binding, where a decrease in hydrodynamic radius would cause the enzyme to diffuse faster (Illien *et al*, 2017; Agudo‐Canalejo *et al*, 2018; Kondrat & Popescu, 2019; Agudo‐Canalejo & Golestanian, 2020). In other instances, it was shown that the measured increase in diffusion can be a result of dissociation of oligomeric enzymes into their subunits (Jee *et al*, 2019). However, neither of these explanations is in line with our hypothesis, since neither implies causality between the rate of Gibbs energy released during catalysis and increased molecular motion.

Yet, as we will illustrate below, there are instances where the Gibbs energy release during catalysis does appear to cause the increase in molecular motion. We do however acknowledge that there is an ongoing discussion about whether increased enzyme diffusion upon catalysis does or does not occur. It has been suggested that experimental artifacts affecting FCS may be responsible for the high diffusion coefficient values reported in the literature (Günther *et al*, 2018; Feng & Gilson, 2019). Along these lines, alternative techniques such as nuclear magnetic resonance (Günther *et al*, 2019), dynamic light scattering (Zhang *et al*, 2018), anti‐Brownian electrokinetic trapping (Chen *et al*, 2020), and single‐molecule displacement mapping (Choi *et al*, 2022) have found no diffusion enhancement for some of these enzymes, namely, aldolase and alkaline phosphatase (Riedel *et al*, 2015; Illien *et al*, 2017). Nevertheless, other techniques, such as single‐molecule measurements using total internal reflection fluorescence microscopy, have reported an even higher diffusion coefficient for urease than the value obtained with FCS (Xu *et al*, 2019). These seemingly contradictory results may be partly explained by differences in the experimental conditions: the enzymatic reaction may have not occurred to the same extent in all experiments, e.g., due to the lack of co‐factors essential for the enzymatic reaction; or if the substrate and product concentrations were different, in which case the Gibbs energy of the reaction would have been different when the measurements were made. Along these lines, a recent paper suggested a correlation between enzyme mobility and the rate of Gibbs energy release (Jee *et al*, 2020).

Despite the uncertainty surrounding the true extent of diffusion enhancement of single enzymes, we still consider it possible that enzymes harness the Gibbs energy released during catalysis to perform work. Here, we illustrate two classes of mechanisms by which this could be accomplished: (i) self‐propulsion of the enzyme or (ii) by the enzyme breaking free from a loose and disordered supramolecular structure.

### Self‐propulsion

**Figure 3:**
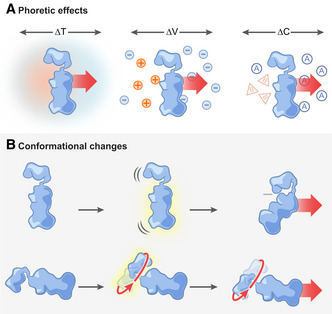
Mechanisms for enzyme self‐propulsion by work performed (A) Phoretic effects, in which an enzyme moves without necessarily having to change its conformation. This motion may be accomplished when local gradients around the enzyme are established during catalysis. These gradients can be of different nature: temperature, ΔT (self‐thermophoresis (Golestanian, 2015), left panel); electrostatic potential, ∆V (self‐electrophoresis (Muddana *et al*, 2010), middle panel), as a result of a differential accumulation of positive (+) and negative (−) charges; or substrate (A) or product (B) concentration, ∆C (self‐diffusiophoresis (Golestanian *et al*, 2005; Banigan & Marko, 2016), right panel). (B) Conformational changes, leading to active swimming motion, either by asymmetric pressure waves across the enzyme (chemoacoustic effect (Riedel *et al*, 2015), top panel), or by directional movement of its structural elements around a fixed point or axis (Slochower & Gilson, 2018) (bottom panel).

Enzyme self‐propulsion could be one consequence of work performed during catalysis potentially resulting in increased molecular motion in the cell. Self‐propulsion provides an active translational component to the enzyme’s purely thermal, stochastic motion (*i.e*., thermal Brownian motion), so that the effective diffusion coefficient increases. Such self‐propulsion can be achieved by phoretic effects (Fig 3A) or conformational changes (Fig 3B). With phoretic effects, enzymes are dragged along a gradient of a relevant thermodynamic parameter such as temperature, electric potential, or species concentration, where the gradient is established by the enzyme’s own catalytic activity. With conformational changes, enzymes alter their shape during catalysis in order to displace the surrounding fluid. In the case of phoretic effects, one would label enzymes as “squirmers”, and those undergoing conformational changes as “swimmers” (Bechinger *et al*, 2016).

Phoretic effects drive molecule motion by the presence of gradients. Such gradients may be externally imposed. For example, ATP gradients have been suggested to be involved in driving the motion of membrane‐bound molecules by phoretic effects (Ramm *et al*, 2021). It is however possible that self‐generated catalysis‐induced gradients, present in the vicinity of an enzyme, could set the enzyme in motion. Importantly, only the latter would account for self‐propulsion. Examples of “self‐phoretic” effects include self‐electrophoresis, self‐diffusiophoresis, and self‐thermophoresis (Golestanian, 2015; Feng & Gilson, 2020) (Fig 3A). Self‐electrophoresis was proposed as a mechanism for the enhanced diffusion of urease (Muddana *et al*, 2010), where the generated ammonium ions would form a local electric field that would generate a piconewton‐scale propulsive force on the enzyme until the ions diffuse away. Yet, this mechanism cannot explain the increased diffusion of enzymes that catalyze reactions involving only neutral species (Feng & Gilson, 2020). Self‐diffusiophoresis of an enzyme would occur by an asymmetric distribution of its reaction products (Golestanian *et al*, 2005), where the gradient is established by the reaction (Banigan & Marko, 2016). Such phoretic motion, however, is dependent on the rather weak interactions between the enzyme, the substrates, and reaction products, rendering this mechanism insufficient to explain the experimental results of increased enzyme diffusion (Feng & Gilson, 2020). Finally, self‐thermophoresis exploits a local temperature gradient, but its effect was found to be fifteen orders of magnitude too low to account for the experimental observations of enhanced diffusion of catalase (Golestanian, 2015).

Alternatively, enzymes may self‐propel by conformational changes causing them to actively swim across the fluid. According to the chemoacoustic model (Riedel *et al*, 2015), the energy released during catalysis by an enzyme with asymmetry between its catalytic site and its center‐of‐mass could generate an asymmetric “pressure wave” that moves across the enzyme and deforms it (Fig 3B, top panel). Upon its deformation, the protein exerts a force on the surrounding fluid, which in turn exerts a force back on the protein, propelling it, and thereby enhancing its apparent diffusion (Riedel *et al*, 2015). The chemoacoustic model has been questioned in later publications, arguing that the heat released by the enzyme‐catalyzed reaction cannot induce a significant acoustic response because of damping by the solvent (Bai & Wolynes, 2015), and that this mechanism would be four orders of magnitude too small to account for the experimental observations (Golestanian, 2015).

Another possibility of how conformational changes during enzyme catalysis could lead to self‐propulsion would be for the enzyme to move some of its domains around fixed points or axes (Fig 3B, bottom panel). Indeed, enzymes can use the released Gibbs energy to drive motion by changing conformational states (Astumian, 2018), with the energy required to drive these conformational changes being comparable to that released during the catalytic reaction (Boehr *et al*, 2010). Thus, it can be envisioned that the Gibbs energy released during an enzyme’s reaction potentially provides the energy required to let structural elements of the enzyme move in a way that results in its self‐propulsion.

However, micro‐/nanoscopic swimmers, such as enzymes, are faced with restrictions on their motion. In fact, for micro‐/nano‐sized swimmers, inertial forces are negligible compared with the viscous forces imposed by the surrounding fluid. This dominance of the viscous forces leads to a remarkable consequence: objects can only “swim” (achieving a non‐zero net displacement) in Newtonian fluids, such as water, if they change their conformation in a non‐reciprocal way (*i.e*., with no time‐reversal symmetry) (Purcell, 1977). This is also the case in the cytoplasm, where molecules are surrounded by water: on very short timescales, they move as though they were in a dilute solution (Di Rienzo *et al*, 2014; Makuch *et al*, 2020). As enzymes are chiral molecules, they will likely undergo such non‐reciprocal conformational changes (Slochower & Gilson, 2018), and there is thus the possibility for their net‐displacement by “swimming” (Bai & Wolynes, 2015).

While swimming by non‐reciprocal conformational changes resembles a physically sound mechanism, some authors argued that the magnitude of diffusion enhancement to be expected from this mechanism is too low to explain the experimental values (Bai & Wolynes, 2015; Golestanian, 2015). Nevertheless, in the highly crowded intracellular environment, the high concentration of enzymes which might swim in this manner still renders an enhanced overall diffusion possible by means of a collective effect. The small hydrodynamic flow generated by each enzyme’s conformational change can add up to a measurable increase in the mean diffusion coefficient, as suggested by Brownian dynamics simulations (Skóra *et al*, 2021).

### Breaking free from a supramolecular structure

**Figure 4:**
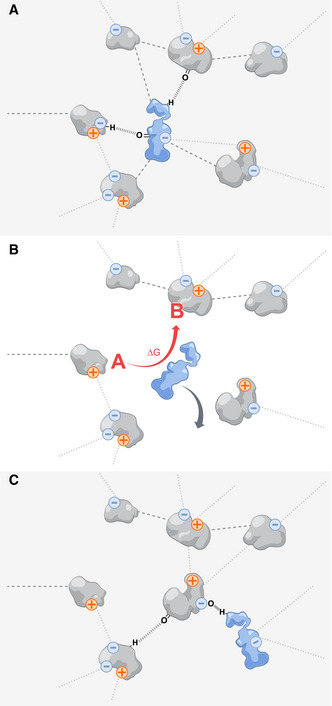
Work performed by an enzyme can lead to its breaking free from a supramolecular structure (A) Due to the high concentration of macromolecules in the cell, proteins can establish multivalent interactions with each other and with other molecules. These interactions can arise between charged (electrostatic interactions, dotted lines), or uncharged parts of each molecule (dashed lines); hydrogen bonds may also be established (parallel dashed lines). (B) Such interactions can be counteracted by the release of Gibbs energy in enzymatic reactions, which allow the enzymes to momentarily escape the supramolecular structure and undergo unhindered diffusive motion. (C) When the energy is dissipated to its surroundings, the protein re‐attaches to the supramolecular structure.

There is a second possible explanation for how enzymes could increase their motion in the cytoplasm upon catalytic action. The cytoplasm is a highly crowded environment (Ellis, 2001; McGuffee & Elcock, 2010), where the short distance between molecules facilitates attractive and repulsive interactions between them (Monteith *et al*, 2015; Yu *et al*, 2016). This proximity, and the establishment of transient multivalent interactions, increases the structural complexity of the cytoplasm, effectively creating a loose and disordered supramolecular structure of proteins and other macromolecules (Fig 4A). For example, enzymes, despite not being structural proteins, can be part of supramolecular structures in both prokaryotes and eukaryotes (Noree *et al*, 2019; Park & Horton, 2019).

With this picture in mind, one can envision that the Gibbs energy released during enzyme catalysis can be used to “break the enzyme free” from such a structure (Fig 4B) until it is once again “integrated” (Fig 4C). This may happen if the Gibbs energy released in the reaction exceeds the energy of the interactions between the enzyme and its neighbors, which is likely since non‐specific protein‐protein interactions are weak (Yu *et al*, 2016). Upon its release from the influence of surrounding macromolecules, an enzyme can undergo unhindered diffusive motion (in agreement with high time‐resolution raster image correlation spectroscopy measurements of protein diffusion (Di Rienzo *et al*, 2014)) until it encounters some other molecules, with which it again interacts and to which it binds. The release of the enzyme from such structures, made possible by the performance of work enabled by the Gibbs energy released during catalysis, can constitute another way of increasing the apparent diffusion of enzymes *in vivo*. This may likewise provide one possible explanation for the observed fluidization of the cytoplasm (Parry *et al*, 2014; Nishizawa *et al*, 2017). Multiple enzymes collectively engaging in their catalytic activity would compromise the integrity of this supramolecular structure, which would otherwise contribute to the glass‐like properties of the cytoplasm.

The two mechanisms discussed above, by which Gibbs energy released during an enzymatic reaction could perform work, manifest as increased metabolism‐dependent intracellular diffusion. Even though the role of self‐propulsion as the cause of enhanced enzyme diffusion observed in *in vitro* experiments has been questioned (Günther *et al*, 2018; Feng & Gilson, 2019), this might be different in the highly crowded environment of the cytoplasm. Even a small increase in enzyme motion could potentially have a significant impact on overall intracellular motion and could thereby explain the *in vivo* observations. Indeed, several theoretical and computational studies have shown that the concerted action of many enzymes undergoing conformational changes can lead to enhancement of the diffusion of passive tracer particles (Mikhailov & Kapral, 2015; Kapral & Mikhailov, 2016; Dennison *et al*, 2017; Hosaka *et al*, 2020), and at least one study showed that in solutions of active urease and aldolase, tracer microspheres experienced an increase in mobility (Zhao *et al*, 2017). According to further studies and in line with the idea of a “stirred up” cytoplasm, a generalized increase in diffusion (not just of tracers, but also of the enzymes themselves) is likewise to be expected (Mikhailov & Kapral, 2015; Kapral & Mikhailov, 2016; Koyano *et al*, 2019; Oyama *et al*, 2019; Skóra *et al*, 2021). We do not know whether it is self‐propulsion or breaking free from a supramolecular structure that explains the catalysis‐dependent enhanced enzyme diffusion in cells. One mechanism might apply for some enzymes, and the other for others. It is also possible that both mechanisms occur simultaneously.

## What are the consequences of increased molecular motion for the cell?

If, as hypothesized above, Gibbs energy released during an enzymatic reaction is transduced into work and leads to increased molecular motion in cells, this might have consequences for a wide range of cellular processes. In general, molecular motion is important for biomolecular functions, as it allows molecules to interact. However, this motion is strongly reduced when the fluidizing activity of metabolism is inhibited and the cytoplasm transitions into a glass‐like state (Parry *et al*, 2014; Nishizawa *et al*, 2017). As such, a catalysis‐induced increase in molecular motion may be important for living cells, for example, for psychrophilic organisms, which inhabit low‐temperature environments and thus need to increase the fluidity of their cytoplasm and membranes (D’Amico *et al*, 2006). For such organisms, catalytic activity would help keeping the cytoplasm fluidized.

Metabolism‐induced molecular motion may also control phase separation, a phenomenon which has shown to influence regulatory processes (Alberti, 2017; Klosin *et al*, 2020; Azaldegui *et al*, 2021). Indeed, studies in colloidal physics showed that actively moving particles tend to cluster (Cates & Tailleur, 2015; Agudo‐Canalejo & Golestanian, 2019; Deblais *et al*, 2020). It is unclear whether the same can be expected from highly mobile enzymes, not only due to the high complexity of molecular interactions in the cell (Söding *et al*, 2020) but also due to other phenomena, such as coalescence of phase‐separated droplets and molecule release from the interface of such droplets (Ranganathan & Shakhnovich, 2020). While it remains unclear if catalysis‐induced molecular motion increases or decreases molecular clustering or phase separation, it seems likely that it will affect it in one way or the other.

Increased molecular motion can also promote structural changes in proteins and other biomolecules. As recently shown, albumin adsorbed to a layer of synthetic molecular motors underwent denaturation when the motors were set into motion (Zhou *et al*, 2020). Similarly, signal transduction and regulatory processes can be perturbed by motion. Molecular movement was suggested to influence signal transduction by mechanically perturbing cytoskeletal elements, which have been speculated to play a role in intracellular signaling (Forgacs *et al*, 2004). Furthermore, as shown *in vitro*, the rate of DNA‐loop formation by the *lac* operon in *E. coli* doubled when the DNA molecules were forced into oscillations (Chen *et al*, 2010). One can conceive that increased molecular motion in the cytoplasm may have similar effects on DNA‐loop formation.

Structural changes of biomolecules such as RNA or proteins, potentially induced by altered molecular motion caused by altered metabolic activity, could also affect regulation. Interestingly, changes in the secondary structure of RNAs were found in *Bacillus subtilis* upon the modification of the metabolic state (Ritchey *et al*, 2020). As another example, the rpoH transcript of *E. coli* undergoes temperature‐induced conformational changes that determine whether it is translated or not (Yuzawa *et al*, 1993). The protein resulting from this translation is a transcription factor, σ^32^, involved in the heat shock response. Notably, σ^32^ regulates CreB (Nonaka *et al*, 2006), and CreB, in turn, activates the enzymes Pta and AckA (Avison *et al*, 2001). These enzymes are responsible for acetate production and “overflow metabolism”, the type of metabolism associated with the identified limit on cellular Gibbs energy dissipation rate (Niebel *et al*, 2019). Beyond transcriptional and translational control, the regulation of enzyme clustering is an opportunity for the cell to exert some control over its metabolism (Sweetlove & Fernie, 2018), particularly at branch points of its metabolic pathways (Castellana *et al*, 2014; Hinzpeter *et al*, 2019). It is thus tempting to think that, if the stability of these clusters is affected by molecular motion, this would be another way for molecular motion to impact regulation in the cell.

While increased molecular movement can be beneficial for some cellular processes and play a role in regulation, we postulate that *excessive* intracellular motion can be detrimental for cells. The latter is not self‐evident, and further investigations are required to examine if it holds true. Still, some ideas could point in this direction. As an example, excessive intracellular motion might lead to protein unfolding. If this is the case, a critical limit on intracellular motion can emerge once the negative effects of increased motion (e.g., protein unfolding) cannot be counteracted anymore (e.g., by chaperones). As another example, molecular motion inside a compartment has been suggested to influence the translocation of macromolecules to/from that compartment, on the basis of 2D‐Langevin dynamics simulation results (Tan *et al*, 2021). Target‐search processes such as transcription factors finding their target DNA‐binding site or pairing of homologous DNA strands constitute an equally interesting example. On the one hand, this search is generally regarded to be sped up by a higher diffusion. On the other hand, dynamic changes in the conformation of the DNA molecules (e.g., unlooping, mentioned above) may lead to an increase in the search time (Felipe *et al*, 2021). Thus, excessive molecular motion might not be compatible with life. As such, a critical limit on molecular motion, induced by work of enzymes, could explain the identified upper limit on the Gibbs energy dissipation rate (Niebel *et al*, 2019).

## Conclusion and outlook

**Figure 5:**
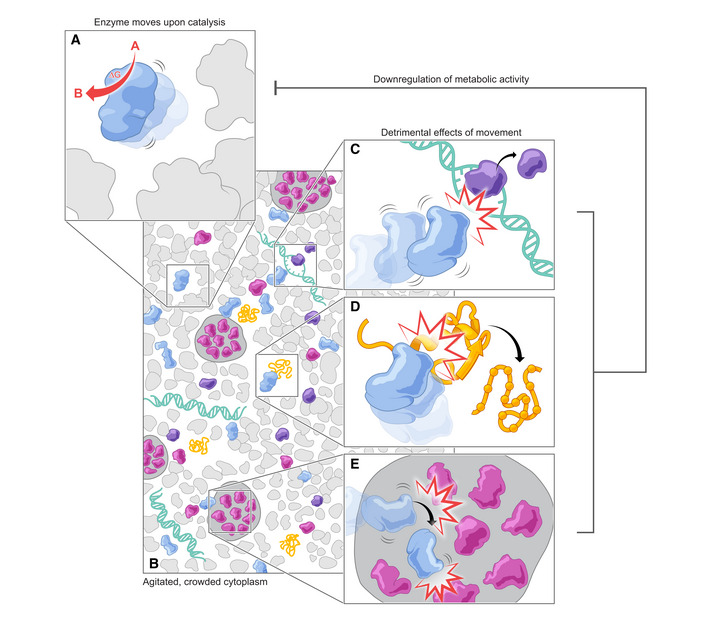
Proposed explanation for the mechanistic basis of the observed limit on the cellular Gibbs energy dissipation rate (A) In metabolically active cells, enzyme catalysis leads to the release of Gibbs energy and results in an increase in enzyme motion. (B) Since the intracellular environment (and cytoplasm, in particular) is highly crowded, collisions between enzymes and other molecules will take place. (C) This increase in motion and collisions impacts various biomolecular functions, for example, transcription. (D) Increased motion can be detrimental for protein folding. (E) Phase separation may be affected by increased molecular motion. Together with other effects, this may ultimately regulate the cell’s metabolic activity, as depicted by the gray negative feedback arrow. The regulation of metabolic activity is expected to control the Gibbs energy dissipation rate, *g^diss^*, and maintain it below its upper limit (Niebel *et al*, 2019).

In summary, we propose that Gibbs energy released during catalysis can be harnessed by enzymes in the form of work, which will ultimately lead to an enhancement of their effective diffusion (Fig 5A). We envision that this increased motion might result from self‐propulsion or from enzymes transiently escaping the influence of neighboring molecules, *i.e*., breaking free from a supramolecular structure. We also expect that excessively increased molecular motion in the cell will on the whole have a detrimental effect on biomolecular functions (Fig 5B–E), which ultimately imposes an upper limit on the Gibbs energy dissipation rate.

At the moment, several key questions remain open (summarized in Table 2). Yet, the hypothesis we outline here by combining evidence from different fields, may explain aspects that still remain controversial within a narrower field. For example, the claim that self‐propulsion explains enhanced enzyme diffusion *in vitro* is still debated (Günther *et al*, 2018; Feng & Gilson, 2019). Could it be that in some experiments the reaction did not occur as expected? Could it be that the key to enhanced enzyme diffusion is the amount of Gibbs energy released? And could it be that in experiments with different outcome, different substrate or product concentrations were used, leading to different ∆Gs? Our perspective might similarly have the potential to resolve the controversy over intracellular temperature measurements. While studies with molecular thermosensors repeatedly reported increased intracellular “temperature” values (Chrétien *et al*, 2018), theoretical considerations have dismissed significant temperature increase inside cells as unrealistically high and suggested that the measurements are confounded by experimental artifacts (Baffou *et al*, 2014; Lane, 2018). Nevertheless, these measurements may be indicative of real phenomena. The reason why molecular thermosensors, which are possibly sensitive to *non‐*equilibrium molecular motion, report increased “temperature” values may be increased molecular motion, as it has been observed in metabolically active cells (Parry *et al*, 2014). Such molecular thermosensors might in fact act as motion sensors.

**Table 2:** Open questions.

Question	How to address
Can the effect of enhanced enzyme diffusion be confirmed and could conflicting claims be explained by differences in activity and/or thermodynamic potential (*i.e*., rate of Gibbs energy dissipation) between experiments?	Full control over biochemical aspects of enzyme diffusion measurements (by FCS, DLS, etc.) and complement such measurements by assessing reactant/product concentrations, the reaction rate, and estimates of Gibbs energy of reaction during the analysis
Is diffusion enhancement dependent on the rate of Gibbs energy dissipation?	Measure enzyme diffusion as a function of the actual Gibbs energy dissipation rate during the experiment
By which mechanisms is increased enzyme motion achieved?	Quantum‐mechanics/molecular dynamics simulations could be used together with experimental techniques (such as high‐precision optical tweezers) to assess dynamic conformational changes of different enzymes undergoing catalysis. High time resolution measurements of enzyme diffusion in cellular environments and novel methods for probing transient protein‐protein interactions
Does the degree of molecular motion in cells correlate with the cellular Gibbs energy dissipation rate?	Measure intracellular motion using different probes (of various length scales) under conditions in which the metabolic activity, and thus the cellular Gibbs energy dissipation rate, has been carefully tuned
Can we understand the molecular structure of the cytoplasm as an “active bath” and what is the influence of catalytically active enzymes on this structure?	Explore the diffusivity changes in the cell and phase separation phenomena upon sudden inactivation and re‐activation of enzymes (e.g., by optogenetics) that dissipate Gibbs energy at different rates
Which regulatory mechanisms controlling metabolism are susceptible to enhanced intracellular motion?	Explore critical regulatory steps in the cell, particularly those that have been previously linked with the response to increased temperature. Assess mRNA and protein conformation under different metabolic conditions, making use of high‐throughput techniques to map *in vivo* structural changes of macromolecules
Is catalysis‐induced increased enzyme motion the cause for the limit on the cellular Gibbs energy dissipation rate, hence the cause for the puzzling metabolic phenotype called “aerobic glycolysis”, “Crabtree effect”, or “overflow metabolism”?	The combined work on the aforementioned aspects will demonstrate whether indeed an active metabolism with enzyme catalysis increasing molecular motion in the cell can explain the inferred limit on the Gibbs energy dissipation rate. Further demonstrating that intracellular motion is dependent on metabolic activity in several organisms and predictive of the onset of changes in metabolic phenotype will show its generality

Questions that still need to be answered, and some ideas on how this may be achieved.

While catalysis‐induced motion may be necessary for cells to accomplish a variety of biomolecular functions, we argue that there is a limit on how much of such motion cells can withstand. As a result, molecular motion should be kept under control, likely by means of a feedback loop acting on metabolic activity (Fig 5A–E). Such a feedback loop would have to sense the changes in molecular motion by resorting to some mechanically sensitive structures (Milstein & Meiners, 2011) that directly or indirectly regulate the expression and/or activity of metabolic enzymes. Following this line of thought, an increase in Gibbs energy dissipation rate driven, e.g., by an increase in substrate uptake rate, would increase molecular motion in the cytoplasm. The result of increased motion on transcription factor binding to DNA, mRNAs, and protein folding, etc., would be such that metabolic activity (and thus, the rate of Gibbs energy dissipation) is capped. In addition to mechanisms sensing molecular motion, sensing of metabolic fluxes (Kochanowski *et al*, 2013) may also play an important role. Through such mechanisms, the cell would reach the upper limit on Gibbs dissipation rate, and cytoplasmic motion be maintained within viable boundaries.

As with any hypothesis, the validity of the ideas we present in this Perspective will have to be tested. A starting point will be to confirm whether intracellular motion correlates with the cellular Gibbs energy dissipation rate. For this to be feasible experimentally, the metabolic conditions must be carefully tuned so that cells operate at a particular cellular Gibbs energy dissipation rate. Then, the motion of differently sized, endogenous or exogenous probes needs to be determined, ideally by various techniques. Another important element is to further demonstrate that enzymes indeed show enhanced motion as a result of their catalytic activity, and to decipher the experimental parameters when it occurs and when not. The mechanisms by which such enhanced motion is achieved should also be clarified: does it occur by self‐propulsion, by breaking free from a supramolecular structure, or by a combination of both? Here, high‐precision optical tweezers may open the possibility to investigate conformational changes that enzymes undergo during catalysis. Experimental evidence is also required to support the second tier of our hypothesis postulating that motion exerts a regulatory effect on metabolism. In that regard, it will be important to assess if the conformation of relevant biomolecules such as mRNA and proteins undergoes changes when exposed to different levels of intracellular motion, potentially harnessing recent technical advances to determine RNA or protein structures *in vivo* (Cappelletti *et al*, 2021; Marinus *et al*, 2021). Importantly, a causal relationship between these conformational changes and changes in metabolic activity and Gibbs energy dissipation needs to be confirmed.

If proven correct, the proposed mechanistic basis for the existing upper limit on Gibbs energy dissipation rate could move our understanding of cellular metabolism and cell functioning to a new level, across different scales. The ideas presented here could build up momentum for “physics of life”, where concepts from physics are used to gain insight into biological and biochemical phenomena. Recent examples include studies on the physicochemistry of cells and its importance for biomolecular processes (Parry *et al*, 2014; Joyner *et al*, 2016; Munder *et al*, 2016; Delarue *et al*, 2018; Persson *et al*, 2020; Xiang *et al*, 2021), phase separation (Alberti & Dormann, 2019; Klosin *et al*, 2020; Fritsch *et al*, 2021) or “active matter” (Battle *et al*, 2016; Gompper *et al*, 2020), which highlight that we are moving towards an exciting, physics‐based understanding of biology. At the same time, and more importantly, this article also offers a new perspective on how physical principles may lead to low‐level regulatory mechanisms in the cell that act in addition to the well‐established control systems that have been described in recent decades. We hope that the perspective that we put forward here will spark new thoughts and ideas, as well pave the way for new research avenues.

## Disclosure and competing interests statement

The authors declare that they have no conflict of interest.

## Supporting information


