# Supplementary material for: Perspective: a stirring role for metabolism in cells
Source: Mol Syst Biol. 2022 Apr 1;18(4):e10822. doi: 10.15252/msb.202110822 (PMC8972047; doi:10.15252/msb.202110822)
Supplement: Supplementary file 1 [file MSB-18-e10822-s001.pdf]

molecular systems biology

# Perspective: A stirring role for metabolism in cells

José Losa, Simeon Leupold, Diego Alonso-Martinez, Petteri Vainikka, Sebastian Thallmair, Katarzyna Tych, Siewert-Jan Marrink, and Matthias Heinemann
**DOI: 10.15252/msb.202110822**

*Corresponding author(s): Matthias Heinemann (m.heinemann@rug.nl)*



## Review Timeline:

| | |
|---|---|
| Submission Date: | 19th Nov 21 |
| Editorial Decision: | 17th Feb 22 |
| Revision Received: | 25th Feb 22 |
| Editorial Decision: | 4th Mar 22 |
| Revision Received: | 5th Mar 22 |
| Accepted: | 9th Mar 22 |

*Editor: Maria Polychronidou*


## Transaction Report:

(Note: With the exception of the correction of typographical or spelling errors that could be a source of ambiguity, letters and reports are not edited. Depending on transfer agreements, referee reports obtained elsewhere may or may not be included in this compilation. Referee reports are anonymous unless the Referee chooses to sign their reports.)

RE: MSB-2021-10822: Perspective: A stirring role for metabolism in cells

Thank you again for submitting this Review/Perspective. I would like to apologise for the exceptional delay in getting back to you. Unfortunately, reviewer #1 did not return any comments after numerous reminders. As we did not want to base our decision on a single report, we invited an additional reviewer (#3). We have now received both reports. As you will see below, both referees acknowledge that the discussed ideas are interesting and thought-provoking. However, they recommend some edits and we would ask you to revise the manuscript accordingly.

Without repeating all the points raised, the more substantial issues raise are the following:

- Reviewer #2 mentions that substantial additional discussion regarding the upper limit of Gibbs free energy dissipation rate needs to be included.

- Reviewer #2 mentions that a more balanced discussion of the possible effects of increased molecular motion is warranted.

- Reviewer #3 indicates that the piece would benefit from a discussion of how the upper limit on the cellular Gibbs energy dissipation rate relates to alternative explanations.

The reviewers make several other constructive suggestions on how to improve the manuscript. I have not yet made specific edits myself, as the text will change during revision. I will send you my edits (if any) after you have made the changes summarized above and those mentioned in the referee reports.

--------------------------------------------------------------------------------

Reviewer #2:

In this perspective manuscript entitled "A stirring role for metabolism in cells", Losa et al. propose a possible explanation for an upper limit of the dissipation rate of cellular Gibbs free energy. Such a limit had previously been extracted from quantitative metabolome and physiological data (Ref. 9). Support for the relevance of this upper limit came from the finding that its integration as a constraint in flux-balance calculations enabled several correct predictions for the physiology and metabolic states of E. coli and S. cerevisiae under different growth conditions. In the current manuscript the authors propose that (i) the Gibbs energy released by metabolic reactions could result in a significant increase of molecular motion within cells ("stirring"), and (ii) this stirring could have a negative impact on biomolecular functions, leading to a negative feedback that would limit the total dissipation rate. The authors do not present any new evidence supporting either part (i) or (ii) of their proposal, but they review pertinent existing data and theoretical results, in particular for part (i), which is closely connected to ongoing research on enhanced diffusion of enzymes that catalyze exothermic reactions. As far as I can tell, part (ii) of the proposal is entirely novel and there is currently not much data to support or invalidate (ii). The systems-level questions raised here are interesting and the manuscript is thought-provoking overall. As such, the manuscript could be suitable as a Perspective article in MSB, depending on the precise criteria that the editors have for this format. I have the following comments and questions for the authors to consider:

1. In the beginning of the manuscript, the authors discuss the core phenomenon that motivates this manuscript: the upper limit on the rate of Gibbs free energy dissipation of the cell. However, considering that this phenomenon provides the starting point of the arguments that follow, this discussion feels too brief and somewhat superficial. For instance, what exactly does the plateau value on the y-axis of the graph in Fig. 1A (rightmost panel) mean and what does it depend on? Is this value proportional to the volume of the cell or is it a rate per volume? What exactly is contained in this total cellular rate of Gibbs free energy dissipation, and what is not contained? In the caption of Fig. 1 it says that cells have to tune down their metabolic activity in the plateau region. What does that mean, and how is a tuned down metabolic activity compatible with increased growth rate, i.e. increased rate of protein biosynthesis. Does the transport of nutrients into the cell play any role here, e.g., in the sense that there is also a limit on how many nutrient molecules per unit time can be imported? How does the plateau value in Fig. 1A change when key regulators or enzymes are over- or under-expressed? Or is the plateau value insensitive to such mutations? It seems essential to discuss such questions about the phenomenon itself prior to discussing possible explanations for the phenomenon - otherwise, the critical reader cannot think about any alternative explanations. For instance, one might think about possible fundamental limitations that would cap the rate of Gibbs free energy dissipation, such as perhaps limited import flux due to limited cell surface for transporters etc - but thinking about such limitations first requires a precise understanding of the experimental observation to be explained.

2. Most of the manuscript is devoted to part (i) of the proposal, which is reasonable, since there are a lot of data and theoretical to discuss in this context. I found this part generally very well done and enjoyable to read (I am familiar with parts of the literature that the authors refer to). The only wish I had at some places was that the authors would make somewhat deeper and stronger statements about the scientific content of some of the literature they review (since this is a perspective article rather than just a regular review). For instance, on page 8, where they mention the theoretical results from Ref. 36 and Ref. 37, there is one sentence devoted to each of these, but as a reader I am left wondering what really is the scientific basis of the statements made here, i.e., what is the core mechanism whereby even a small change in the diffusion coefficient of proteins may lead to significant changes in the fluidization state of the cytoplasm, and why would the binding of mRNA to the ribosome require a temperature of more than 1000 Kelvin?

Minor note: On page 7, the text at the end of "Box 1" is cut off in the pdf I received.

3. At the end of page 10 the authors refer to the possibility that enzyme self-propulsion can be due to phoretic effects where gradients of relevant thermodynamic parameters are self-generated by the enzymes. However, phoretic forces (Ref. 56) and thermodynamic forces (Ref. 54 and Mohajerani et al., Biochemistry, 2008) due to non self-generated substrate gradients have also been suggested to cause enzyme chemotaxis and/or antichemotaxis. As gradients of intracellular components are often present in cells (e.g. Erin Chen Y. et al., PNAS, 2011; Timothy E. Saunders et al., Developmental Cell, 2012; Doogie Oh et al., PNAS, 2016), it seems relevant to contrast the cases of self-generated vs. induced enzyme propulsion.

4. The authors suggest that the Gibbs free energy released by chemical reactions can be used by enzymes to break free from

supramolecular structures. However, in a sense the opposite effect can also occur (reaction-induced accumulation of enzymes): If the motion of enzymes is phoretically driven, it has been shown that enzymes of different types could form static or even dynamic supramolecular aggregates (Agudo-Canalejo & Golestanian, PRL, 2019). Also, non-even enzyme distributions could spontaneously form through a feedback between the enzyme and its substrate (Giunta et al., Communications Physics, 2020).

5. In the section "How could enzymes perform work during catalysis?", the authors mention that reactant and solvent molecules can experience increased mobility upon catalysis. In this context the authors could also refer to the study by Ortiz-Rivera et al, PNAS, 2016 where it is shown that the reaction of immobilized enzymes (urease) can cause the formation of convective flows in a synthetic biological setup due to the exothermicity of the reaction combined with changes of the solution composition due to the reaction. Also, recently it has been shown that the activity of urease in phase-separated droplets can induce flow through a combination of effects due to a sustained pH gradient (Testa A., et al., Nature Communications, 2021). PH gradients therefore could also play a role in intracellular "stirring".

6. The authors refer to Ref. 49 in the context of FCS measurements of solutions of purified enzymes mixed with their substrate. In Ref. 49 however the enzymes under considerations are attached to a phospo-lipid membrane. Consider using this reference in the context of membrane-bound enzymes. Related to this, the recent study by Song S. et al, Nature Communications, 2021 shows that spontaneous symmetry breaking in the organization of freely diffusing surface-bound catalase and urease leads to self-propulsion of coacervates.

7. Regarding part (ii) of the proposal, I did not find the authors argument very convincing, since it was mostly based on assumed effects that could equally well also go in the opposite direction. For instance, in the second paragraph of page 17 the authors suggest that signal transduction and regulatory processes could be perturbed by an increased intracellular motion. However, target search processes within cells (e.g. protein-DNA target search), which are part of signal transduction, could be accelerated by the stirring. Generally, I do acknowledge that too much stirring may be detrimental - intuitively, this even seems likely at some point - but the crucial question are how much stirring is too much and could enzyme activity ever push the cell into that regime? Ultimately, this will have to be resolved experimentally, as the authors also discuss in their Conclusion and Outlook section.

Reviewer #3:

In this perspective article, Losa et al build upon their previous work suggesting that there is an upper limit in the cellular Gibbs energy dissipation rate that determines important metabolic phenotypes such as overflow metabolism. Here, the authors discuss the potential mechanisms and consequences of such a limit in energy dissipation rate. In particular, the authors propose that enzymatic activity in a cell causes a collective increase in intracellular enzyme motion which acts as a global negative feedback mechanism to regulate metabolic activity.

Overall, this article is very well written with figures that nicely illustrate the authors' main points. Although the authors pulled together recent evidence from an impressive range of scientific fields, both the overall logic as well as the individual arguments are easy to follow for readers who are not deeply immersed in the physics of life (such as this reviewer). I particularly appreciate the authors' efforts to suggest experiments that could answer some of the outstanding questions, and the balanced discussion of contradictory evidence. Moreover, the article's theme is very much in line with recent efforts to elucidate the principles of metabolic regulation using physics-based approaches. Thus, I believe that this article will be of great interest for the readership of MSB, similar to landmark perspectives previously published in MSB (such as Molenaar et al, 2009).

I only have one criticism: the authors' arguments are built on upon the premise that there is indeed an upper limit on the cellular Gibbs energy dissipation rate which ultimately determines metabolic phenotypes such as overflow metabolism. The authors justify their focus by pointing out in the introduction that this postulated mechanism provides excellent predictions of experimentally observed metabolic phenotypes (i.e. onset of overflow metabolism). However, recent works have put forward alternative explanations for frequently observed growth-rate dependent patterns of metabolic operation, most notably a study from Terence Hwa's laboratory suggesting that the onset of overflow metabolism is determined by the growth-rate dependent reallocation of proteome resources (PMID 26632588). This study and others (e.g. from the laboratory of Bas Teusink, PMID 33821549) provide similarly good predictions of experimentally observed metabolic phenotypes without invoking any limits on the cellular Gibbs energy dissipation rate, leaving me to wonder about the cause-and-effect relationship between proteome allocation and energy dissipation rate (e.g. maybe the observed limit on energy dissipation rate is simply a consequence of proteome allocation constraints). I argue this perspective would benefit from including a brief discussion of how the authors' proposed mechanism relates to such alternative explanations (for example in a separate box). It is well possible that these different mechanisms are fully compatible with each other, and I believe that the authors are very well positioned to provide some much-needed clarity to the field.

## Editorial remarks and suggestions

**Thank you again for submitting this Review/Perspective. I would like to apologise for the exceptional delay in getting back to you. Unfortunately, reviewer #1 did not return any comments after numerous reminders. As we did not want to base our decision on a single report, we invited an additional reviewer (#3). We have now received both reports. As you will see below, both referees acknowledge that the discussed ideas are interesting and thought-provoking. However, they recommend some edits and we would ask you to revise the manuscript accordingly.**

**Without repeating all the points raised, the more substantial issues raise are the following:**

**- Reviewer #2 mentions that substantial additional discussion regarding the upper limit of Gibbs free energy dissipation rate needs to be included.**

We have added the requested information to the main text and the caption of the respective figure (Fig. 1).

**- Reviewer #2 mentions that a more balanced discussion of the possible effects of increased molecular motion is warranted.**

Following reviewer #2's comments, we have now further developed the discussion on the potential effect of molecular motion on target search processes, introducing one relevant reference.

**- Reviewer #3 indicates that the piece would benefit from a discussion of how the upper limit on the cellular Gibbs energy dissipation rate relates to alternative explanations.**

We have done this by adding a new box (Box 1) where we now discuss these alternative explanations.

**The reviewers make several other constructive suggestions on how to improve the manuscript. I have not yet made specific edits myself, as the text will change during revision. I will send you my edits (if any) after you have made the changes summarized above and those mentioned in the referee reports.**

**Moreover:**

**- For Reviews/Perspectives we typically do not have supplementary information. Figure S1 can be provided as a normal figure.**

We made a simplified version of this earlier supplementary figure and added it as a figure to the box (Fig. 2 in the new version of the manuscript).

**- The References should be formatted according to the reference style of Molecular Systems Biology.**

Done.

**- The figures should be provided as separate files (one file per figure).**

Done.

## Reviewer #2:

**In this perspective manuscript entitled "A stirring role for metabolism in cells", Losa et al. propose a possible explanation for an upper limit of the dissipation rate of cellular Gibbs free energy. Such a limit had previously been extracted from quantitative metabolome and physiological data (Ref. 9). Support for the relevance of this upper limit came from the finding that its integration as a constraint in flux-balance calculations enabled several correct predictions for the physiology and metabolic states of E. coli and S. cerevisiae under different growth conditions. In the current manuscript the authors propose that (i) the Gibbs energy released by metabolic reactions could result in a significant increase of molecular motion within cells ("stirring"), and (ii) this stirring could have a negative impact on biomolecular functions, leading to a negative feedback that would limit the total dissipation rate. The authors do not present any new evidence supporting either part (i) or (ii) of their proposal, but they review pertinent existing data and theoretical results, in particular for part (i), which is closely connected to ongoing research on enhanced diffusion of enzymes that catalyze exothermic reactions. As far as I can tell, part (ii) of the proposal is entirely novel and there is currently not much data to support or invalidate (ii). The systems-level questions raised here are interesting and the manuscript is thought-provoking overall. As such, the manuscript could be suitable as a Perspective article in MSB, depending on the precise criteria that the editors have for this format. I have the following comments and questions for the authors to consider:**

We would like to thank the reviewer for the excellent summary and the kind words.

**1. In the beginning of the manuscript, the authors discuss the core phenomenon that motivates this manuscript: the upper limit on the rate of Gibbs free energy dissipation of the cell. However, considering that this phenomenon provides the starting point of the arguments that follow, this discussion feels too brief and somewhat superficial. For instance, what exactly does the plateau value on the y-axis of the graph in Fig. 1A (rightmost panel) mean and what does it depend on? Is this value proportional to the volume of the cell or is it a rate per volume? What exactly is contained in this total cellular rate of Gibbs free energy dissipation, and what is not contained? In the caption of Fig. 1 it says that cells have to tune down their metabolic activity in the plateau region. What does that mean, and how is a tuned down metabolic activity compatible with increased growth rate, i.e. increased rate of protein biosynthesis. Does the transport of nutrients into the cell play any role here, e.g., in the sense that there is also a limit on how many nutrient molecules per unit time can be imported? How does the plateau value in Fig. 1A change when key regulators or enzymes are over- or under-expressed? Or is the plateau value insensitive to such mutations? It seems essential to discuss such questions about the phenomenon itself prior to discussing possible explanations for the phenomenon - otherwise, the critical reader cannot think about any alternative explanations. For instance, one might think about possible fundamental limitations that would cap the rate of Gibbs free energy dissipation, such as perhaps limited import flux due to limited cell surface for transporters etc - but thinking about such limitations first requires a precise understanding of the experimental observation to be explained.**

We have addressed the reviewer’s concerns. Specifically, we have clarified that the Gibbs energy dissipation rate is, in the way that we have defined it, a parameter that is normalized by the cell dry weight. If cell size (or mass) were the major determinant of the limit of Gibbs energy dissipation rate, then one would expect the latter value to be increase for smaller-sized cells. This could be the case, if - as the reviewer rightly alluded to -, nutrient uptake is limited by the surface-to-volume ratio of the cell.

However, the values that we obtained in our previous work (-4.9 and -3.7 $kJ.gDW^{-1}.h^{-1}$, for *E. coli* and *S. cerevisiae*, respectively; Niebel et al. 2019) were much too close. Note that these two organisms differ by more than one order of magnitude in volume (and similarly in terms of dry weight). This has been addressed in l.48-53.

The reviewer rightly called our attention to the caption of Fig. 1, where we mention that metabolic activity is “tuned down” when the limit of Gibbs energy dissipation rate is reached. This was admittedly a poor choice of words from our side. We have since reformulated the sentence, now mentioning that the cell must *redistribute its metabolic fluxes* towards less dissipative reactions, thus leaving the door open for further increase in growth rate.

As for the effect of potential over-/under-expression of certain key proteins on the limit of Gibbs energy dissipation rate, we have to acknowledge that, for the time being, we cannot claim whether this limit will remain unaltered or not. Training the sort of models which we presented in our previous work (Niebel et al. 2019) – a process from which yields the value of the upper limit - requires highly accurate physiological data. We currently do not have data that would allow us to obtain a reliable estimate of the said limit.

Agreeing with the reviewer’s call for presenting the reader with alternative explanations, we have decided to include an additional box which compares our thermodynamics-based model with others that rely on alternative physical constraints.

**2. Most of the manuscript is devoted to part (i) of the proposal, which is reasonable, since there are a lot of data and theoretical to discuss in this context. I found this part generally very well done and enjoyable to read (I am familiar with parts of the literature that the authors refer to). The only wish I had at some places was that the authors would make somewhat deeper and stronger statements about the scientific content of some of the literature they review (since this is a perspective article rather than just a regular review). For instance, on page 8, where they mention the theoretical results from Ref. 36 and Ref. 37, there is one sentence devoted to each of these, but as a reader I am left wondering what really is the scientific basis of the statements made here, i.e., what is the core mechanism whereby even a small change in the diffusion coefficient of proteins may lead to significant changes in the fluidization state of the cytoplasm, and why would the binding of mRNA to the ribosome require a temperature of more than 1000 Kelvin?**

Thank you for this comment. We agree that more details should have been provided in places where references are made. Therefore, not only have we added more “scientific content” to the specific cases that the reviewer mentioned here (l.234-244), but also at several other places in the manuscript (namely: l. 263; 273; 381; 399; 422)

**Minor note: On page 7, the text at the end of "Box 1" is cut off in the pdf I received.**

This formatting issue has been corrected in the new version of the manuscript.

**3. At the end of page 10 the authors refer to the possibility that enzyme self-propulsion can be due to phoretic effects where gradients of relevant thermodynamic parameters are self-generated by the enzymes. However, phoretic forces (Ref. 56) and thermodynamic forces (Ref. 54 and Mohajerani et al., Biochemistry, 2008) due to non self-generated substrate gradients have also been suggested to cause enzyme chemotaxis and/or antichemotaxis. As gradients of intracellular components are often**

**present in cells (e.g. Erin Chen Y. et al., PNAS, 2011; Timothy E. Saunders et al., Developmental Cell, 2012; Doogie Oh et al., PNAS, 2016), it seems relevant to contrast the cases of self-generated vs. induced enzyme propulsion.**

We agree with the relevance of contrasting self-generated with external gradients. Although this "opposition" was present in the previous version of the manuscript ("Phoretic effects drive motion of molecules by the presence of gradients. While such gradients may be externally imposed (…)"), we have attempted to make the text clearer, stressing that only self-generated gradients would be relevant in the context of our discussion. We have also added one new reference concerning non-self-generated gradients (Ramm et al. 2021) – but none of the references kindly suggested by the reviewer. The reason being that the former reference establishes a connection between *gradients* of ATP and *mobility* (of membrane-bound molecules), whereas the other references merely describe the existence of intracellular gradients, without an explicit link to motion.

**4. The authors suggest that the Gibbs free energy released by chemical reactions can be used by enzymes to break free from supramolecular structures. However, in a sense the opposite effect can also occur (reaction-induced accumulation of enzymes): If the motion of enzymes is phoretically driven, it has been shown that enzymes of different types could form static or even dynamic supramolecular aggregates (Agudo-Canalejo & Golestanian, PRL, 2019). Also, non-even enzyme distributions could spontaneously form through a feedback between the enzyme and its substrate (Giunta et al., Communications Physics, 2020).**

The reviewer is right to suggest that increased motion could also lead to local accumulation of enzymes. This is something that we mention further down in the text, i.e. in the paragraph dedicated to phase separation (l. 445). As such, the first of the two references suggested by the reviewer was conveniently used to further support one of our previous statements. As for the second reference, although certainly a remarkable and interesting work, we do not see a good way of introducing it in our text without disturbing the flow of ideas, thus having opted to leave it out.

**5. In the section "How could enzymes perform work during catalysis?", the authors mention that reactant and solvent molecules can experience increased mobility upon catalysis. In this context the authors could also refer to the study by Ortiz-Rivera et al, PNAS, 2016 where it is shown that the reaction of immobilized enzymes (urease) can cause the formation of convective flows in a synthetic biological setup due to the exothermicity of the reaction combined with changes of the solution composition due to the reaction. Also, recently it has been shown that the activity of urease in phase-separated droplets can induce flow through a combination of effects due to a sustained pH gradient (Testa A., et al., Nature Communications, 2021). PH gradients therefore could also play a role in intracellular "stirring".**

We appreciate that the reviewer suggested these additional references. We have included them in the text. For the sake of maintaining readability, we did not do so in the paragraph pointed out by the reviewer, but rather in the paragraph that immediately precedes it (l. 247).

**6. The authors refer to Ref. 49 in the context of FCS measurements of solutions of purified enzymes mixed with their substrate. In Ref. 49 however the enzymes under considerations are attached to a phospo-lipid membrane. Consider using this reference in the context of membrane-bound enzymes. Related to this, the recent study by Song S. et al, Nature Communications, 2021 shows that**

**spontaneous symmetry breaking in the organization of freely diffusing surface-bound catalase and urease leads to self-propulsion of coacervates.**

We thank the reviewer for noting that one of the references (Ghosh et al. 2019) was not cited in the most accurate way. Following the suggestion, we now explicitly discriminate between the the two types of experimental studies (with free enzymes, and with membrane-bound enzymes; l. 268), and included the new reference (Song et al. 2021) in the latter (l.273)

**7. Regarding part (ii) of the proposal, I did not find the authors argument very convincing, since it was mostly based on assumed effects that could equally well also go in the opposite direction. For instance, in the second paragraph of page 17 the authors suggest that signal transduction and regulatory processes could be perturbed by an increased intracellular motion. However, target search processes within cells (e.g. protein-DNA target search), which are part of signal transduction, could be accelerated by the stirring. Generally, I do acknowledge that too much stirring may be detrimental - intuitively, this even seems likely at some point - but the crucial question are how much stirring is too much and could enzyme activity ever push the cell into that regime? Ultimately, this will have to be resolved experimentally, as the authors also discuss in their Conclusion and Outlook section.**

Indeed, the current lack of decisive experimental evidence makes it difficult to support our suggestion that too much motion is detrimental to the cell. As the reviewer also acknowledges, work still needs to be done on this front to clarify whether this is the case and in fact our perspective also aims to provide new ideas for future research. The reviewer mentions the particular example of target search as one which could benefit from increased motion, as search time would likely decrease. Yet, even here it is not so clear-cut: it is also possible that increased motion leads to a decrease in the efficiency of target search (hence, increase in search time), as suggested by the modeling study of Felipe et al. (2021) – see Fig.7 therein. This point was also added to the discussion (l. 489).

## Reviewer #3:

**In this perspective article, Losa et al build upon their previous work suggesting that there is an upper limit in the cellular Gibbs energy dissipation rate that determines important metabolic phenotypes such as overflow metabolism. Here, the authors discuss the potential mechanisms and consequences of such a limit in energy dissipation rate. In particular, the authors propose that enzymatic activity in a cell causes a collective increase in intracellular enzyme motion which acts as a global negative feedback mechanism to regulate metabolic activity.**

**Overall, this article is very well written with figures that nicely illustrate the authors' main points. Although the authors pulled together recent evidence from an impressive range of scientific fields, both the overall logic as well as the individual arguments are easy to follow for readers who are not deeply immersed in the physics of life (such as this reviewer). I particularly appreciate the authors' efforts to suggest experiments that could answer some of the outstanding questions, and the balanced discussion of contradictory evidence. Moreover, the article's theme is very much in line with recent efforts to elucidate the principles of metabolic regulation using physics-based approaches. Thus, I believe that this article will be of great interest for the readership of MSB, similar to landmark perspectives previously published in MSB (such as Molenaar et al, 2009).**

Thanks a lot for the kind words. We hope that in a couple of years our perspective will indeed be seen as forward-looking as the -in the meantime- seminal 2009 MSB article by Molenaar, Teusink and colleagues.

**I only have one criticism: the authors' arguments are built on upon the premise that there is indeed an upper limit on the cellular Gibbs energy dissipation rate which ultimately determines metabolic phenotypes such as overflow metabolism. The authors justify their focus by pointing out in the introduction that this postulated mechanism provides excellent predictions of experimentally observed metabolic phenotypes (i.e. onset of overflow metabolism). However, recent works have put forward alternative explanations for frequently observed growth-rate dependent patterns of metabolic operation, most notably a study from Terence Hwa's laboratory suggesting that the onset of overflow metabolism is determined by the growth-rate dependent reallocation of proteome resources (PMID 26632588). This study and others (e.g. from the laboratory of Bas Teusink, PMID 33821549) provide similarly good predictions of experimentally observed metabolic phenotypes without invoking any limits on the cellular Gibbs energy dissipation rate, leaving me to wonder about the cause-and-effect relationship between proteome allocation and energy dissipation rate (e.g. maybe the observed limit on energy dissipation rate is simply a consequence of proteome allocation constraints). I argue this perspective would benefit from including a brief discussion of how the authors' proposed mechanism relates to such alternative explanations (for example in a separate box). It is well possible that these different mechanisms are fully compatible with each other, and I believe that the authors are very well positioned to provide some much-needed clarity to the field.**

We agree that by applying growth-rate dependent reallocation of proteome resources in FBA models, excellent predictions can similarly be obtained. The nice point is that our thermodynamics-based FBA approach and the protein allocation-based FBA mathematically both boil down to the same constraint, namely one that caps a weighted sum of fluxes, where in our approach the weights are the drGs, and in the protein allocation approach the weights are typically a $k_{cat}$/enzyme mass.

The protein allocation approach is very intuitive and we think that this is part of the reason why it is currently the favored approach by researchers in the field. Our thermodynamics-based approach, resting on a limit in the cellular Gibbs energy dissipation rate, is much more challenging to explain mechanistically (i.e. much less intuitive). Indeed, our perspective article is an effort to provide a mechanistic explanation for the limit in the cellular Gibbs energy dissipation rate. The future will show how these things all fit together; potentially they are just two sides of the same coin.

In any case, we agree with the reviewer that it would be good to mention the protein allocation constraint as an alternative, and to discuss the differences and similarities of both FBA approaches. We do this now in a separate box (Box 1). We would like to thank the reviewer for this very useful suggestion.

4th Mar 2022

RE: MSB-2021-10822R, Perspective: A stirring role for metabolism in cells

Thank you for sending us the revised article. I think that the edits have addressed the concerns and suggestions of the reviewers.

I have made some suggestions for text edits (see attached .doc file). Please let me know if you are OK with them and feel free to correct anything that you are not on board with.
